# Identification and Characterization of *trans*-Isopentenyl Diphosphate Synthases Involved in Herbivory-Induced Volatile Terpene Formation in *Populus trichocarpa*

**DOI:** 10.3390/molecules24132408

**Published:** 2019-06-29

**Authors:** Nathalie D. Lackus, Nora P. Petersen, Raimund Nagel, Axel Schmidt, Sandra Irmisch, Jonathan Gershenzon, Tobias G. Köllner

**Affiliations:** 1 Max Planck Institute for Chemical Ecology, Department of Biochemistry, Hans-Knöll-Strasse 8, 07745 Jena, Germany; 2 Iowa State University, Roy J. Carver Department of Biochemistry, Biophysics and Molecular Biology, 4254 Molecular Biology Building, 2437 Pammel Drive, Ames, IA 50011, USA; 3 Michael Smith Laboratories, University of British Columbia, Vancouver, BC V6T 1Z4, Canada

**Keywords:** *Populus trichocarpa*, isoprenyl diphosphate synthase, prenyl transferase, monoterpene, sesquiterpene, volatile organic compound (VOC), *Lymantria dispar*

## Abstract

In response to insect herbivory, poplar releases a blend of volatiles that plays important roles in plant defense. Although the volatile bouquet is highly complex and comprises several classes of compounds, it is dominated by mono- and sesquiterpenes. The most common precursors for mono- and sesquiterpenes, geranyl diphosphate (GPP) and (*E,E*)-farnesyl diphosphate (FPP), respectively, are in general produced by homodimeric or heterodimeric *trans*-isopentenyl diphosphate synthases (*trans*-IDSs) that belong to the family of prenyltransferases. To understand the molecular basis of herbivory-induced terpene formation in poplar, we investigated the *trans-IDS* gene family in the western balsam poplar *Populus trichocarpa*. Sequence comparisons suggested that this species possesses a single FPP synthase gene (*PtFPPS1*) and four genes encoding two large subunits (*PtGPPS1.LSU* and *PtGPPS2.LSU*) and two small subunits (*PtGPPS.SSU1* and *PtGPPS.SSU2*) of GPP synthases. Transcript accumulation of *PtGPPS1.LSU* and *PtGPPS.SSU1* was significantly upregulated upon leaf herbivory, while the expression of *PtFPPS1*, *PtGPPS2.LSU*, and *PtGPPS.SSU2* was not influenced by the herbivore treatment. Heterologous expression and biochemical characterization of recombinant PtFPPS1, PtGPPS1.LSU, and PtGPPS2.LSU confirmed their respective IDS activities. Recombinant PtGPPS.SSU1 and PtGPPS.SSU2, however, had no enzymatic activity on their own, but PtGPPS.SSU1 enhanced the GPP synthase activities of PtGPPS1.LSU and PtGPPS2.LSU in vitro. Altogether, our data suggest that PtGPPS1.LSU and PtGPPS2.LSU in combination with PtGPPS.SSU1 may provide the substrate for herbivory-induced monoterpene formation in *P. trichocarpa*. The sole FPP synthase PtFPPS1 likely produces FPP for both primary and specialized metabolism in this plant species.

## 1. Introduction

Plant specialized metabolites provide protection against herbivores not only by acting as toxins and feeding deterrents but also by serving as defensive signals. Many plant species, for example, emit complex volatile blends upon herbivory that can attract herbivore enemies [1] or signal impending danger to distant parts of the same plant or neighboring plant [2]. Herbivory-induced plant volatile blends are often complex and possess dozens of substances from diverse classes of natural compounds, including terpenes, green leaf volatiles, aromatic compounds, and nitrogen-containing amino acid derivatives [3]. Although their qualitative and quantitative composition differs between plant species, herbivory-induced volatile blends are often dominated by monoterpenes and sesquiterpenes [1].

One of the most important enzyme groups in plant terpene biosynthesis is the *trans*-isoprenyl diphosphate synthases (*trans*-IDSs), which belong to the family of prenyl transferases [4,5,6]. IDS enzymes catalyze condensations of the basic C_5_ terpene building blocks, isopentenyl diphosphate (IPP) and dimethylallyl diphosphate (DMAPP), to generate C_10_, C_15_, C_20_, and larger prenyl diphosphates [4,7,8]. These monoterpene, sesquiterpene, and diterpene precursors, geranyl diphosphate (GPP), (*E,E*)-farnesyl diphosphate (FPP), and (*E,E,E*)-geranylgeranyl diphosphate (GGPP), respectively, are produced by short-chain *trans*-IDS enzymes that are designated as geranyl diphosphate synthases (GPPSs), farnesyl diphosphate synthases (FPPSs), and geranylgeranyl diphosphate synthases (GGPPSs) according to their major product. Longer prenyl diphosphates such as geranylfarnesyl diphosphate (GFPP) or solanesyl diphosphate are formed by medium-chain and long-chain IDSs [9].

Crystal structures of *trans*-IDS proteins from bacteria, fungi, animals, and plants have revealed a common tertiary structure consisting of 13 α-helices [10,11,12,13,14]. Ten of these α-helices surround a large central cavity and the size and geometric structure of this pocket are thought to be critical for product specificity, with large amino acids at the bottom blocking further chain elongation of the prenyl diphosphate in the active site [7]. The entrance to the pocket is flanked by two highly conserved DD(xx)_2/4_D motifs named the first aspartate-rich motif (FARM) and the second aspartate-rich motif (SARM) [10]. The aspartate residues of the FARM and SARM motifs were shown to bind divalent metal ions such as magnesium (Mg^2+^) or manganese (Mn^2+^) that in turn bind the diphosphate of the homo-allylic substrate [15].

In plants, *trans*-IDSs usually occur as dimers or as tetramers [14]. While FPPSs and GGPPSs act solely as homodimers, GPPSs can be active both as homo- and heterodimers. The heterodimers consist of a large subunit (LSU) possessing the two DD(xx)_2/4_D motifs required for catalysis and a catalytically inactive small subunit (SSU) that modulates the catalytic efficiency and chain length specificity of the protein complex [16,17,18,19]. The amino acid motif CxxxC, which is located upstream of the FARM motif and highly conserved among the small and large subunits of heterodimeric plant GPPSs, has been shown to play a critical role in the physical interactions between the two subunits [17].

In previous studies, we investigated the formation and ecological functions of herbivory-induced volatiles emitted from different poplar species [20,21,22,23,24]. The western balsam poplar (*Populus trichocarpa*), for example, releases more than 50 volatiles when damaged by gypsy moth larvae (*Lymantria dispar*) [20]. Some of the emitted compounds have been shown to deter attacking caterpillars, while others are attractive to a generalist parasitoid [22,24]. The most prominent components of the *P. trichocarpa* volatile blend are the monoterpene (*E*)-β-ocimene and the sesquiterpene (*E,E*)-α-farnesene, which together comprise more than 60% of the total herbivory-induced volatile emission [20]. Terpene synthases (TPS) able to convert the terpene precursors GPP and FPP into monoterpenes and sesquiterpenes, respectively, have been identified in *P. trichocarpa* and gene expression analysis showed that they are significantly upregulated upon herbivory [20,21,25]. Whether poplar *trans*-IDS enzymes also control the formation of terpene volatiles by providing increased pools of GPP and FPP upon herbivory is unknown.

To understand the contribution of *trans*-IDSs to the formation of herbivory-induced monoterpenes and sesquiterpenes in poplar, we investigated putative GPPS and FPPS enzymes in the poplar model species *P. trichocarpa*. Heterologous expression in *Escherichia coli* and enzyme assays with purified recombinant proteins were performed to functionally characterize the identified candidates. Analysis of *IDS* gene expression by RNAseq and quantitative real-time PCR showed that the formation of GPP is likely catalyzed by two GPPSs and increases upon herbivory, while FPP is constitutively produced by a single FPPS in *P. trichocarpa* leaves.

## 2. Results

### 2.1. The trans-IDS Gene Family in P. trichocarpa Possesses a Single FPPS Gene and Four Putative GPPS Genes

**Figure 1:**
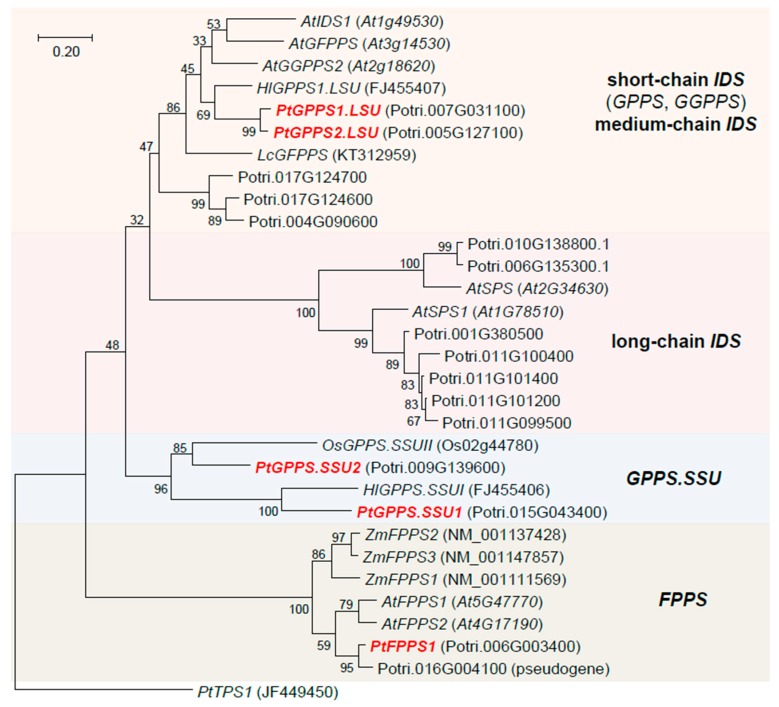
Rooted tree of putative *IDS* genes of *Populus trichocarpa* together with a selection of characterized *IDS* genes from other plant species. Bootstrap values (*n* = 1000 replicates) are shown next to the branches. The tree is drawn to scale, with branch lengths in units of base substitutions per site. Genes characterized in this study are shown in bold and red. The *P. trichocarpa* terpene synthase gene *PtTPS1* was used as outgroup. Accession numbers (NCBI) or gene identifiers are given in parentheses.

A BLAST analysis showed that *P. trichocarpa* possesses 16 genes with high sequence similarity to *trans*-*IDS* genes from other plants (Figure 1). According to their position in a phylogenetic tree of plant *trans*-*IDS* genes, two of these candidates (Potri.006g003400 and Potri.016g004100) were considered as putative *FPPS* genes. However, since the protein encoded by Potri.016g004100 lacked about 160 amino acids including the catalytically important FARM motif (Figure S1), the gene was assumed to be a pseudogene. The remaining putative *FPPS* gene Potri.006g003400 was named *PtFPPS1*. Two other genes, Potri.007g031100 and Potri.005g127100, showed similarity to a GPPS large subunit from hops and were designated as *PtGPPS1.LSU* and *PtGPPS2.LSU*, respectively. Two putative GPPS small subunits Potri.015g043400 and Potri.009g139600 grouped together with the catalytically inactive small subunits of GPPSs from hops and rice and so were named *PtGPPS.SSU1* and *PtGPPS.SSU2*, respectively. The remaining ten *IDS* candidates were classified as *GGPPS* genes, medium-chain *IDS* genes, or long-chain *IDS* genes based on their sequence similarity to respective *IDS* genes from other plants (Figure 1). Because they are not involved in volatile terpene formation, they were not considered further in this study.

In general, the monoterpene precursor GPP is produced in plastids and the sesquiterpene precursor FPP is produced in the cytosol [26]. A signal peptide prediction showed that the putative GPPS proteins PtGPPS1.LSU, PtGPPS2.LSU, PtGPPS.SSU1, and PtGPPS.SSU2 indeed contained N-terminal signal peptides that likely target the proteins to plastids (Figure S2). PtFPPS1, however, had no signal peptide and is therefore presumably located in the cytosol. The characteristic FARM and SARM motifs required for catalytic activity could be identified in PtFPPS1, PtGPPS1.LSU, PtGPPS2.LSU, and PtGPPS.SSU2 (Figure S1). Both motifs were absent in the small subunit PtGPPS.SSU1. The CxxxC motif was present in all small and large GPPS subunits but not in PtFPPS1. Notably, PtGPPS.SSU2 contained a second CxxxC motif located upstream of the SARM motif (Figure S1).

### 2.2. PtGPPS1.LSU and PtGPPS.SSU1 are Induced Upon Herbivory

**Figure 2:**
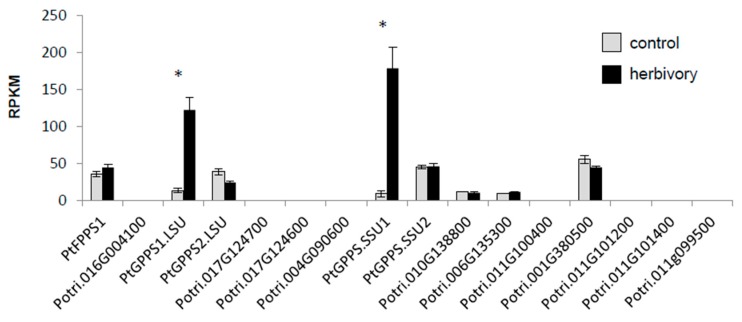
Transcript accumulation of putative *IDS* genes in *Lymantria dispar*-damaged and undamaged *Populus trichocarpa* leaves. Gene expression in herbivore-damaged (herbivory) and undamaged (control) leaves was analyzed by Illumina sequencing and mapping the reads to the transcripts of the *P. trichocarpa* genome version v3.0. Expression was normalized to RPKM (reads per kilobase of transcript per million mapped reads). Significant differences in EDGE tests (*p* ≤ 0.001) are visualized by asterisks. Means ± SE are shown (n = 4 biological replicates). *PtFPPS1* (*p* = 0.10443, weighted difference (WD) = 1.61411 × 10^−5^); Potri.016g004100 (*p* = 1, WD = 9.19891 × 10^−8^); *PtGPPS1.LSU* (*p* = 5.45471 × 10^−27^, WD = 0.000173475); *PtGPPS2.LSU* (*p* = 0.008505317, WD = −2.06885 × 10^−5^); Potri.017g124700 (*p* = 1, WD = 6.61109 × 10^−9^); Potri.017g124600 (*p* = 1, WD = −1.12331 × 10^−8^); Potri.004g090600 (*p* = 0.500021071, WD = 7.53238 × 10^−7^); *PtGPPS.SSU1* (*p* = 5.0039 × 10^−28^, WD = 0.000269403); *PtGPPS.SSU2* (*p* = 0.667703669, WD = 4.49444 × 10^−6^); Potri.010g138800 (*p* = 0.918085163, WD = −1.05663 × 10^−6^); Potri.006g135300 (*p* = 0.338295043, WD = 3.93657 × 10^−6^); Potri.011g100400 (*p* = 1, WD = 0); Potri.001g380500 (*p* = 0.253763889, WD = −1.31158× 10^−5^); Potri.011g101200 (*p* = 1, WD = 1.12627 × 10^−7^); Potri.011g101400 (*p* = 1, WD = 1.04422 × 10^−7^); Potri.011g099500, no expression detected.

The analysis of RNAseq data obtained from gypsy moth larvae-damaged and undamaged *P. trichocarpa* leaves [27] revealed that transcript accumulation of *PtGPPS1.LSU* and *PtGPPS.SSU1* was significantly increased in herbivore-damaged leaves in comparison to undamaged control leaves (Figure 2). The expression of *PtFPPS1*, *PtGPPS2.LSU*, and *PtGPPS.SSU2*, however, was not influenced by the herbivory treatment. Three of the putative long-chain *IDS* genes, Potri.010g138800, Potri.006g135300, and Potri.001g380500, also showed no changes in transcript accumulation upon herbivory, while the remaining *IDS* genes including the putative *FPPS* pseudogene Potri.016g004100 were not or nearly not expressed in this organ (Figure 2).

A qRT-PCR analysis with *PtFPPS1*, *PtGPPS1.LSU*, *PtGPPS2.LSU*, *PtGPPS.SSU1*, and *PtGPPS.SSU2* confirmed the herbivory-induced upregulation of *PtGPPS1.LSU* and *PtGPPS.SSU1* gene expression (Figure S3).

### 2.3. PtFPPS1 Produces FPP While PtGPPS1.LSU and PtGPPS2.LSU Have GPPS and GGPPS Activity In Vitro

**Figure 3:**
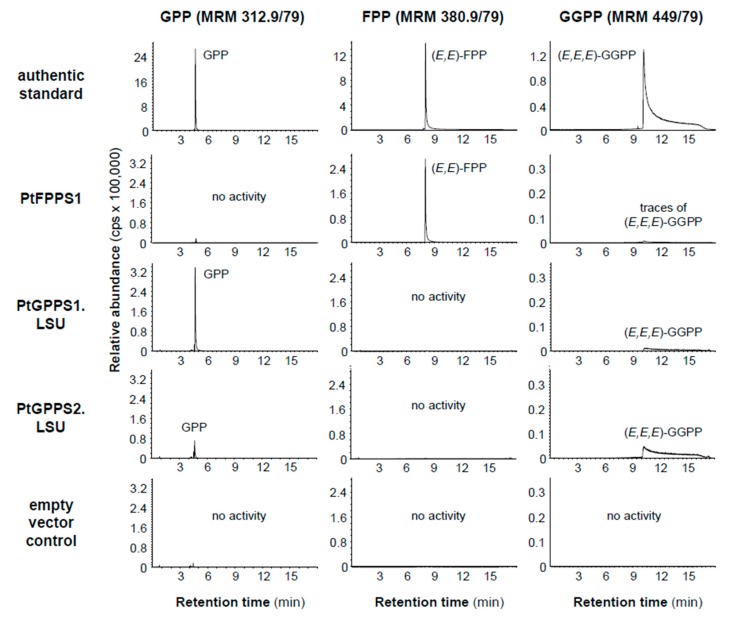
IDS activity of PtFPPS1, PtGPPS1.LSU, and PtGPPS2.LSU. The genes were heterologously expressed in *Escherichia coli* and purified recombinant proteins were incubated with the substrates IPP and DMAPP. Products were analyzed using LC-MS/MS and identified with authentic standards. The empty expression vector was expressed under the same conditions, assayed with IPP and DMAPP, and used as negative control.

**Figure 4:**
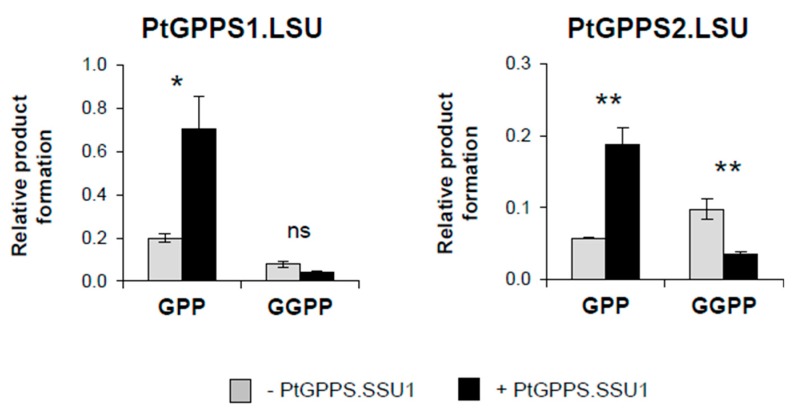
The small subunit PtGPPS.SSU1 enhances the GPPS activity of PtGPPS1.LSU and PtGPPS2.LSU. Purified recombinant PtGPPS1.LSU and PtGPPS2.LSU were incubated with IPP and DMAPP either in the presence (black bars) or absence (grey bars) of purified recombinant PtGPPS.SSU1. Product formation was analyzed by LC-MS/MS and quantified using authentic standards. Means ± SE of triplicate assays are shown. Asterisks indicate statistical significance in Student’s t-tests and from Mann–Whitney Rank Sum Tests, respectively. PtGPPS1.LSU ± PtGPPS.SSU1 GPP (*p* = 0.010, t = −4.067); PtGPPS1.LSU ± PtGPPS.SSU1 GGPP (*p* = 0.075, t = 2.241); PtGPPS2.LSU ± PtGPPS.SSU1 GPP (*p* = 0.003, t = −5.379); PtGPPS2.LSU ± PtGPPS.SSU1 GGPP (*p* = 0.009, t = 4.091).

The full-length open reading frame (ORF) of PtFPPS1 and the N-terminal truncated ORFs of PtGPPS1.LSU (Δ71), PtGPPS2.LSU (Δ72), PtGPPS.SSU1 (Δ41), and PtGPPS.SSU2 (Δ45) lacking the predicted signal peptide sequences were cloned into a bacterial expression vector and heterologously expressed in *Escherichia coli*. Purified recombinant proteins were incubated with the potential substrates IPP and DMAPP and product formation was analyzed using liquid chromatography–tandem mass spectrometry (LC-MS/MS). The enzyme assays revealed that PtFPPS1 catalyzes the condensation of IPP and DMAPP to FPP and traces of GGPP (Figure 3). PtGPPS1.LSU and PtGPPS2.LSU, however, produced both GPP and GGPP, but no FPP. Under the conditions in our assays, the GPPS activity of PtGPPS1.LSU was higher in comparison to its GGPPS activity, while PtGPPS2.LSU showed the opposite behavior and produced more GGPP than GPP (Figure 4). The two GPPS small subunits PtGPPS.SSU1 and PtGPPS.SSU2 had no activity when assayed with IPP and DMAPP (Figure S4).

### 2.4. The Small Subunit PtGPPS.SSU1 Enhances the GPPS Activity of PtGPPS1.LSU and PtGPPS2.LSU In Vitro

It has been reported that small subunits of heterodimeric GPPS complexes can influence the activity and product specificity of the large GPPS subunits [14,16]. Thus, we tested the effect of PtGPPS.SSU1 and PtGPPS.SSU2 on the catalytic activity of PtGPPS1.LSU and PtGPPS2.LSU. Assays containing equal amounts of purified recombinant PtGPPS1.LSU and PtGPPS.SSU1 showed higher GPPS activity and lower GGPPS activity in comparison to assays containing only the large subunit PtGPPS1.LSU (Figure 4). The product specificity of PtGPPS2.LSU was also influenced by PtGPPS.SSU1. While PtGPPS2.LSU produced GGPP as main product when assayed alone, the chain-length specificity was significantly shifted towards increased GPPS activity by PtGPPS.SSU1 (Figure 4). PtGPPS.SSU2, however, did not influence the activity or chain-length specificity of PtGPPS1.LSU and PtGPPS2.LSU in our in vitro assays (Figure S5).

## 3. Discussion

Terpenes represent one of the largest groups of natural compounds and are ubiquitous in almost all kingdoms of life. In plants, terpenes play important roles in defense against insects and pathogens. Poplars, for example, produce volatile terpenes in response to insect herbivory and the released terpenes have been discussed as attractants for herbivore enemies, as antimicrobial compounds, and as inter- and intra-plant defense signals [20,24,25]. To investigate the formation of herbivory-induced terpenes in poplar, we identified and characterized *trans*-IDS enzymes that provide the precursors for mono- and sesquiterpenes in the model poplar species *P. trichocarpa*.

*P. trichocarpa* possesses a mid-sized *trans*-IDS gene family with 16 members, a number similar to that reported from Arabidopsis, which has been shown to contain 17 *trans*-IDS genes [28]. Based on sequence comparisons and dendrogram analysis, the individual poplar genes could be assigned to subfamilies of functionally-related *trans*-IDSs (Figure 1). Two out of the 16 putative *trans*-IDS genes, *PtGPPS1.LSU* and *PtGPPS2.LSU*, grouped together with *GPPS*, *GGPPS*, and *GFPPS* from other plants and were thus considered as potential GPPS genes in this study. Another group of three related poplar genes (Potri.017g124700, Potri.017g124600, and Potri.004g090600) clustered in the same clade of the phylogenetic tree; however, the low bootstrap value for this branching does not support a reliable prediction of enzymatic function. More importantly, Potri.017g124700, Potri.017g124600, and Potri.004g090600 were not expressed in undamaged and herbivore-damaged poplar leaves and so were excluded from the list of candidates potentially involved in herbivore induced mono- and sesquiterpene formation in this organ. The two remaining GPPS candidates PtGPPS1.LSU and PtGPPS2.LSU were similar to the large subunit of a heterodimeric GPPS from hops [17] and produced GPP and GGPP when tested with IPP and DMAPP as substrates (Figure 3). Notably, each protein showed a different product specificity. While PtGPPS1.LSU showed a preference for the formation of GPP, PtGPPS2.LSU formed more GGPP than GPP (Figure 4). Catalytic promiscuity in vitro has been reported for other short-chain IDS enzymes [14,29,30,31,32]. The multifunctional PaIDS1 from Norway spruce, for example, was shown to produce GPP and GGPP when assayed with IPP and DMAPP [31], while PaIDS3 from the same species formed GPP, FPP, and GGPP in vitro [30,31]. However, whether such catalytic promiscuity also occurs in vivo and has biological relevance is still unclear.

While FPPSs and GGPPSs usually function as homodimers, GPPSs can form heterodimers consisting of a large subunit containing all amino acid motifs necessary for catalytic activity and a small subunit that is catalytically inactive [14]. Biochemical characterization of several heterodimeric GPPS complexes in different plants revealed that the small subunits can modulate the activity and chain-length specificity of the enzyme complex [14,16,17,18,33]. The large subunit of hops G(G)PPS, for example, was shown to produce GPP, FPP, and GGPP from IPP and DMAPP in vitro. Coexpression of G(G)PPS with the small subunit GPPS.SSU, however, yielded a heterodimeric complex exhibiting enhanced catalytic formation of GPP [17]. Among the 16 putative *trans*-IDSs identified in *P. trichocarpa*, two (PtGPPS.SSU1 and PtGPPS.SSU2) showed significant sequence similarity to small GPPS subunits from other plants (Figure 1). One of them, *PtGPPS.SSU1*, was found to be induced upon herbivory (Figure 2) and the recombinant protein modified the chain-length specificity of PtGPPS1.LSU towards increased GPPS activity in vitro (Figure 4). Because *PtGPPS1.LSU* was also highly upregulated upon herbivory, we propose that both proteins form a heterodimeric GPPS complex that provides GPP as a precursor for the herbivory-induced formation of monoterpenes in planta. The GPP-forming activity of PtGPPS2.LSU was also enhanced by PtGPPS.SSU1 in vitro and it is thus conceivable that PtGPPS2.LSU-PtGPPS.SSU1 complexes may also contribute to herbivory-induced GPP formation. In the absence of PtGPPS.SSU1 in undamaged leaves, PtGPPS2.LSU by itself could form GGPP and GPP for other metabolic processes. Transcript accumulation of the second small subunit PtGPPS.SSU2 was not influenced by herbivory (Figure 2). In contrast to PtGPPS.SSU1, recombinant PtGPPS.SSU2 did not influence the chain-length specificity of PtGPPS1.LSU and PtGPPS2.LSU in vitro. Notably, PtGPPS.SSU2 possessed both the FARM and the SARM motifs but showed no IDS activity (Figure S1). It is conceivable that PtGPPS.SSU2 functions as an interaction partner for other constitutively expressed *trans*-IDS genes and modulates their activities. The elucidation of its exact role in prenyl diphosphate formation would be a worthwhile aim for future studies.

*FPPS* genes have been identified and characterized in a number of plant species from diverse families. Database surveys and detailed investigations with *Arabidopsis thaliana*, *Zea mays*, *Artemisia tridentata*, and the tree species *Eucommia ulmoides* suggest that plants usually possess small *FPPS* gene families with two to five members [34,35,36,37,38]. The different *FPPS* gene copies of a plant species can be differentially regulated, resulting in distinct spatial and temporal expression patterns [36,39]. The maize *fpps1* gene, for example, is strongly expressed in roots and has been suggested to provide the precursor for the formation of constitutive sesquiterpenes and triterpenes in this organ. Maize *fpps3*, however, is induced upon caterpillar feeding in leaves and likely controls the herbivory-induced production of defense sesquiterpenes [36]. In contrast to maize and other plants, *P. trichocarpa* possesses only one functional *FPPS* gene *PtFPPS1*, which is constitutively expressed in leaves and not induced upon herbivory (Figure 2). Biochemical characterization of purified recombinant PtFPPS1 confirmed its activity as FPPS (Figure 3). Another poplar gene (Potri.016g004100) that grouped together with *PtFPPS1* in the FPPS clade of plant *trans*-IDS genes (Figure 1) was characterized as a pseudogene. Its ORF encodes a protein lacking significant parts of typical FPPS sequences such as the FARM motif essential for catalytic activity (Figure S1). Moreover, RNAseq data as well as our unsuccessful attempts to amplify the ORF from cDNA suggest that Potri.016g004100 is not expressed in poplar leaves (Figure 2). Since no other obvious *FPPS* genes could be identified in the *P. trichocarpa* genome, it is likely that PtFPPS1 acts as the sole FPPS and provides a constitutive pool of FPP for both primary and specialized metabolism. In this scenario, the herbivory-induced formation of volatile sesquiterpenes may be controlled by the upregulation of poplar sesquiterpene synthases as already reported in previous studies [20,21] and/or by an increase of metabolic flux through the mevalonate pathway.

## 4. Material and Methods

### 4.1. Plants and Insects

*Populus trichocarpa* trees were propagated from monoclonal stem cuttings (clone 625, NW-FVA, Hann. Münden, Germany) and grown under summer conditions in the greenhouse (24 °C, 60% rel. humidity, 16 h/8 h light cycle) in a 1:1 mixture of sand and soil (Klasmann potting substrate, Klassmann-Deilmann, Geeste, Germany), until they reached about 1 m in height. Gypsy moth (*Lymantria dispar*) egg batches were kindly provided by Melody Keena (USDA Forest Service, Hamden, CT, USA). After hatching, the caterpillars were reared on an artificial diet (Gypsy moth diet, MP Biomedicals LLC, Illkirch, France) until they reached the third instar.

For the herbivore treatment, trees were enclosed with a PET bag (“Bratschlauch”, Toppits, Minden, Germany) by fixing the ends of the bags to the branches with cable binders. Caterpillars were starved individually for 24 h before they were released on the trees in the bag (50 third instar caterpillars per tree). Caterpillars were allowed to feed for 24 h (17:00–17:00). Leaf material was harvested immediately at the end of the treatment, flash-frozen with liquid nitrogen and stored at −80 °C until further use. Five biological replicates were performed for both the control and herbivory treatments.

### 4.2. RNA Extraction and Reverse Transcription

Total RNA was extracted from frozen and ground leaf material using the InviTrap Spin Plant RNA Mini Kit (STRATEC, Birkenfeld, Germany) according to manufacturer’s instructions. The RNA was analyzed on an Agilent Bioanalyzer 2100 and RNA 6000 Nano Labchip using the Expert software (Agilent version B.02.02.SI258, Santa Clara, CA, USA) to determine quality, integrity, and rRNA ratios. A NanoDrop 2000c (Thermo Fisher Scientific, Erlangen, Germany) was used for RNA quantification. cDNA was synthesized from DNase I-treated (Thermo Fisher Scientific, Erlangen, Germany) total RNA using SuperScript III First-Strand Synthesis SuperMix (Thermo Fisher Scientific, Erlangen, Germany).

### 4.3. Identification and Isolation of trans-IDS Genes

To identify putative *P. trichocarpa trans*-IDS genes, a thorough search of the Phytozome database (http://www.phytozome.net/poplar) was conducted using the amino acid sequences of AtFPPS1 (At5G47770), AtSPS1 (At1G78510), and AtGGPPS2 (At2g18620) from *Arabidopsis thaliana* and HlGPPS.SSUI (FJ455406) from *Humulus lupulus* as an input for BLASTP analyses. One of the resulting sequences was tentatively annotated as FPP synthase (PtFPPS1 (Potri.006g003400)) and four sequences were tentatively annotated as large and small subunits of GPP synthases (PtGPPS1.LSU (Potri.007g031100), PtGPPS2.LSU (Potri.005g127100), PtGPPS.SSU1 (Potri.015g043400), and PtGPPS.SSU2 (Potri.009g139600)).

The full-length ORF of *PtFPPS1* and the N-terminal truncated ORFs of *PtGPPS1.LSU, PtGPPS2.LSU, PtGPPS.SSU1*, and *PtGPPS.SSU2* lacking the putative signal peptides predicted with the programs ChloroP (http://www.cbs.dtu.dk/services/ChloroP/), TargetP (http://www.cbs.dtu.dk/services/TargetP/), and PREDOTAR (https://urgi.versailles.inra.fr/Tools/Predotar) (Figure S2) were amplified from cDNA made from herbivore-damaged *P. trichocarpa* leaves using the primers listed in Table 1 and cloned into the expression vectors pASK-IBA33+ (IBA-GmbH, Göttingen, Germany, *PtGPPS1.LSU, PtGPPS2.LSU, PtGPPS.SSU1*, and *PtGPPS.SSU2*) or pASK-IBA37+ (*PtFPPS1*). The constructs were introduced into the *E. coli* strain TOP10 (Thermo Fisher Scientific, Erlangen, Germany) and fully sequenced to check for amplification errors.

### 4.4. Heterologous Expression and Enzyme Assays

Liquid cultures of bacteria harboring the expression constructs were grown at 18 °C to an OD_600_ of 0.5. Anhydrotetracycline (IBA-GmbH, Göttingen, Germany) was added at a final concentration of 200 µg l^−1^, and the cultures were incubated for 16 h at 18 °C. Cells were collected by centrifugation and disrupted by a 4 × 30 s treatment with a sonicator (Bandelin UW2070, Berlin, Germany) in chilled extraction buffer (50 mM MOPSO, pH 8.0, 500 mM NaCl, 20 mM imidazole, 1% Tween 20, and 10% (*v/v*) glycerol). The debris was separated by centrifugation for 20 min at 16,100 g and 4 °C. The C-terminal His-tagged proteins PtGPPS1.LSU, PtGPPS2.LSU, PtGPPS.SSU1, and PtGPPS.SSU2 as well as the N-terminal His-tagged protein PtFPPS1 were purified using Ni-NTA spin columns (Qiagen, Hilden, Germany) according to the manufacturer’s instructions. The purified proteins were eluted with 50 mM Tris-HCl (pH 8.0) containing 0.5 M NaCl, 250 mM imidazole, and 10% (*v/v*) glycerol and desalted into assay buffer (25 mM MOPSO, pH 7.2, 10% (*v/v*) glycerol) by passage through an Econopac 10DG column (BioRad, Hercules, CA, USA). Enzyme assays were carried out using 6 µg of purified protein, 10 mM MgCl_2_ and 0.1 mM MnCl_2_ as cofactors, and the substrates IPP and DMAPP (each 50 µM) in 100 µL assay buffer at 30 °C for 1 h. Assay results are reported as the mean of at least three independent replicates.

### 4.5. LC-MS/MS Analysis of GPP, FPP, and GGPP

Enzyme products were analyzed using an Agilent 1200 HPLC system (Agilent Technologies, Santa Clara, CA, USA) coupled to an API 3200 triple-quadrupole mass spectrometer (Applied Biosystems, Foster City, CA, USA). For separation, a ZORBAX Extended C-18 column (1.8 μm, 50 mm × 4.6 mm; Agilent Technologies, Santa Clara, CA, USA) was used. The mobile phase consisted of 5 mM ammonium bicarbonate in water as solvent A and acetonitrile as solvent B, with the flow rate set at 0.8 mL/min and the column temperature kept at 20 °C. Separation was achieved by using a gradient starting at 0% B (*v/v*), increasing to 10% B in 2 min, 64% B in 12 min, and 100% B in 2 min (1-min hold), followed by a change to 0% B in 1 min (5-min hold) before the next injection. The injection volume for samples and standards was 3 μL and the autosampler temperature was 4 °C. The mass spectrometer was used in the negative electrospray ionization (EI) mode. Optimal settings were determined using standards. Levels of ion source gases 1 and 2 were set at 60 and 70 psi, respectively, with a temperature of 700 °C. Curtain gas was set at 30 psi and collision gas was set at 7 psi, with all gases being nitrogen. Ion spray voltage was maintained at −4200 V. Multiple-reaction monitoring (MRM) was used to monitor analyte parent ion-to-product ion formation: *m/z* 312.9/79 for GPP, *m/z* 380.9/79 for FPP, and *m/z* 449/79 for GGPP. Data analysis was performed using Analyst Software 1.6 Build 3773 (Applied Biosystems, Foster City, CA, USA).

### 4.6. Gene Expression Analysis (RNAseq and qRT-PCR)

For the analysis of poplar *trans*-IDS gene expression, we reanalyzed the raw data (SRA accession number PRJNA516861) of an RNAseq experiment recently described in Günther et al. [27]. In this experiment, eight transcriptomes of *P. trichocarpa* leaves (four biological replicates of untreated control trees and four biological replicates of gypsy moth-treated trees) were sequenced on an IlluminaHiSeq 2500 sequencer (Max Planck Genome Center, Cologne, Germany) with 18 Mio reads per library (100 base pair, single end). Trimming of the obtained Illumina reads and mapping to the poplar gene model version 3.0 (https://phytozome.jgi.doe.gov/pz/portal.html) were performed with the program CLC Genomics Workbench (Qiagen Bioinformatics, Hilden, Germany) (mapping parameter: length fraction, 0.7; similarity fraction, 0.9; max number of hits, 25). Empirical analysis of digital gene expression (EDGE) implemented in the program CLC Genomics Workbench was used for gene expression analysis.

Qualitative real time polymerase chain reactions (qRT-PCRs) were performed in optical 96-well plates using a Stratagene Mx3000P real-time thermocycler (Stratagene, Carlsbad, CA, USA). Brilliant SYBR Green QPCR Master Mix (Agilent Technologies, Santa Clara, CA, USA) containing a double strand intercalating dye was used to visualize the amplification progress by reading the fluorescence. ROX was used as an internal standard to normalize the fluorescence. As negative controls, samples containing water instead of cDNA were used. Five biological replicates with each three technical replicates were performed. Each reaction contained cDNA and sequence-specific primers (Table 1) in a volume of 25 μL. Primer efficiency was tested using five different dilutions (1- to 64-fold) and calculated with the standard curve method [40]. The thermocycler protocol was performed with an initial denaturing step for 10 min at 95 °C followed by 40 cycles of 30 s with 95 °C, 60 s with 55 °C, and 60 s at 72 °C. For primer efficiency tests, the runs were followed by a melting curve analysis consisting of one cycle with 95 °C for 30 s, 55 °C for 30 s and 95 °C for 30 s. For each primer pair, the amplification products were cloned into the pCR4-TOPO TA vector (Thermo Fisher Scientific, Erlangen, Germany) and sequenced. Amplification was analyzed with the MxPro QPCR Software (Agilent Technologies, Santa Clara, CA, USA). The FPP synthase gene *PtFPPS1* was used as housekeeping gene for the qRT analysis of other *trans*-IDS genes, since the fluctuation of its Ct values (~0,9 Ct) was minimal between control plants and herbivore-treated plants.

### 4.7. Sequence Analysis and Tree Reconstruction

A multiple sequence alignment of identified poplar *trans*-IDS genes and characterized *trans*-IDS genes from other plants was computed using the MUSCLE (codon) algorithm (gap open, −2.9; gap extend, 0; hydrophobicity multiplier, 1.2; clustering method, UPGMB) implemented in MEGA6 [41]. Based on this alignment, a tree was reconstructed with MEGA6 using a maximum likelihood algorithm and the Kimura 2-parameter model. Codon positions included were 1st + 2nd + 3rd + noncoding. All positions with less than 80% site coverage were eliminated. Ambiguous bases were allowed at any position. A bootstrap resampling analysis with 1000 replicates was performed to evaluate the topology of the generated tree.

### 4.8. Statistical Analysis

Throughout the manuscript, data are presented as means ± SE. To compare relative expression of *PtGPPS1.LSU, PtGPPS2.LSU, PtGPPS.SSU1*, and *PtGPPS.SSU2*, in comparison between control and gypsy moth damaged *P. trichocarpa* leaves and the catalytic activity of PtGPPS1.LSU and PtGPPS2.LSU with/without PtGPPS.SSU1, Student’s *t*-tests or Mann–Whitney Rank Sum Tests were performed with SigmaPlot 11.0 for Windows (Systat Software Inc., Erkrath, Germany). Whenever necessary, the data were transformed through exponentiation to meet statistical assumptions such as normality and homogeneity of variances.

### 4.9. Accession Numbers

Sequence data for genes in this article can be found in the GenBank under the following identifiers: *PtFPPS1* (MK492685), *PtGPPS1.LSU* (MK492686), *PtGPPS2.LSU* (MK492687), *PtGPPS.SSU1* (MK492683), and *PtGPPS.SSU2* (MK492684).

## Figures and Tables

**Table 1:** Oligonucleotides used for isolation (cloning) and qRT-PCR analysis (qRT-PCR) of poplar *trans*-IDS genes.

Name	Sequence	Usage
PtFPPS1-fwd	ATGGTAGGTCTCAGCGCATGGCAGATCTGAAGTCAACGTTC	cloning
PtFPPS1-rev	ATGGTAGGTCTCATATCATTTCTGCCTCTTGTAAATTTTAGCC	cloning
PtGPPS1.LSU-fwd	ATGGTAGGTCTCAAATGCCCACTTTTGATTTCAAGTCTTACA	cloning
PtGPPS1.LSU-rev	ATGGTAGGTCTCAGCGCTGTTTTGCCTGTAAGCAATGTAATTAG	cloning
PtGPPS2.LSU-fwd	ATGGTAACCTGCATTAAATGCCCACTTTTGATTTCAAGTCTTATAT	cloning
PtGPPS2.LSU-rev	ATGGTAACCTGCATTAGCGCTGTTTTGCCTGTAAGCAATGTAATTAG	cloning
PtGPPS.SSU1-fwd	ATGGTAGGTCTCAAATGGCAACTTCCAATGGCACTACTTAC	cloning
PtGPPS.SSU1-rev	ATGGTAGGTCTCAGCGCTAACATTGCCGGAAGTGGTCCCT	cloning
PtGPPS.SSU2-fwd	ATGGTAGGTCTCAAATGTCAAAAACACCCCAGTTTGATTTAAA	cloning
PtGPPS.SSU2-rev	ATGGTAGGTCTCAGCGCTACTTGACTCACCAAAACTGAAACC	cloning
PtFPPS1-fwd1	GGTTGCCCAAGGTTGGTCTTATTGC	qRT-PCR
PtFPPS1-rev1	TGAGTAGTAGGCGGTCTTGTACTG	qRT-PCR
PtGPPS1.LSU-fwd1	TGGCGAAAGCTATTGG	qRT-PCR
PtGPPS1.LSU-rev1	CGGTTCCTCCACCTAATATG	qRT-PCR
PtGPPS2.LSU-fwd1	GTTGCTGGACAAGTTGTG	qRT-PCR
PtGPPS2.LSU-rev1	TCCAATACTCCTCGCGTATC	qRT-PCR
PtGPPS.SSU1-fwd1	TGCTCCAGCCTTGTGCATAG	qRT-PCR
PtGPPS.SSU1-rev1	ACCATCCCATCTCCTGTTAG	qRT-PCR
PtGPPS.SSU2-fwd1	GATTGCTAGCCGGTGCCAAG	qRT-PCR
PtGPPS.SSU2-rev1	CCTCCGCTACCTCTATAGCC	qRT-PCR

## Supplementary Materials

The following are available online. Figure S1. Amino acid comparison of PtFPPS1, PtGPPS1.LSU, PtGPPS2.LSU, PtGPPS.SSU1, and PtGPPS.SSU2 with FPPS and GPPS from other plants. The first aspartate-rich motif (FARM), the second aspartate-rich motif (SARM) and the two CxxxC motifs are marked with red boxes. The predicted signal peptides of the poplar IDS proteins are underlined. Figure S2. Signal peptide prediction of PtFPPS1, PtGPPS1.LSU, PtGPPS2.LSU, PtGPPS.SSU1, and PtGPPS.SSU2. Signal peptide (cTP) prediction was done using the web-based prediction programs chlorop v1.1 (http://www.cbs.dtu.dk/services/ChloroP/) (A), targetp v1.1 (http://www.cbs.dtu.dk/services/TargetP/) (B), and Predotar v1.04 (https://urgi.versailles.inra.fr/predotar/) (C). Figure S3. Relative expression of putative *IDS* genes in undamaged (control) and *Lymantria dispar*-damaged (herbivory) leaves of *Populus trichocarpa*. Gene expression was analyzed using quantitative RT-PCR. Means and standard errors are shown (n = 5). Asterisks indicate statistical significance in Student’s t-tests and from Mann-Whitney Rank Sum Tests, respectively. *PtFPPS1* (*P* = 0.151, T = 20.00); *PtGPPS1.LSU* (*P* = 0.008, T = 15.00); *PtGPPS2.LSU* (*P* = 0.329, t = 1.040); *PtGPPS.SSU1* (*P* = 0.008, T = 15.00); *PtGPPS.SSU2* (*P* = 0.796, t = 0.268). Figure S4. PtGPPS.SSU1 and PtGPPS.SSU2 have no IDS activity when tested with IPP and DMAPP. The genes were heterologously expressed in *Escherichia coli* and purified recombinant proteins were incubated with the substrates IPP and DMAPP. Product analysis was done using LC-MS/MS. Enzyme products were identified using authentic standards. An empty vector was expressed under the same conditions, assayed with IPP and DMAPP, and used as negative control. Figure S5. The activity of PtGPPS1.LSU (A) and PtGPPS2.LSU (B) is not influenced by PtGPPS.SSU2. The genes were heterologously expressed in *Escherichia coli* and purified recombinant proteins were incubated with the substrates IPP and DMAPP. Product analysis was done using LC-MS/MS. Enzyme products were identified using authentic standards.
