# Supplementary material for: Identification and Characterization of trans-Isopentenyl Diphosphate Synthases Involved in Herbivory-Induced Volatile Terpene Formation in Populus trichocarpa
Source: Molecules. 2019 Jun 29;24(13):2408. doi: 10.3390/molecules24132408 (PMC6651613; doi:10.3390/molecules24132408)
Supplement: Supplementary file 1 [file molecules-24-02408-s001.zip › molecules-527043-supplementary.pptx]

## Slide 1
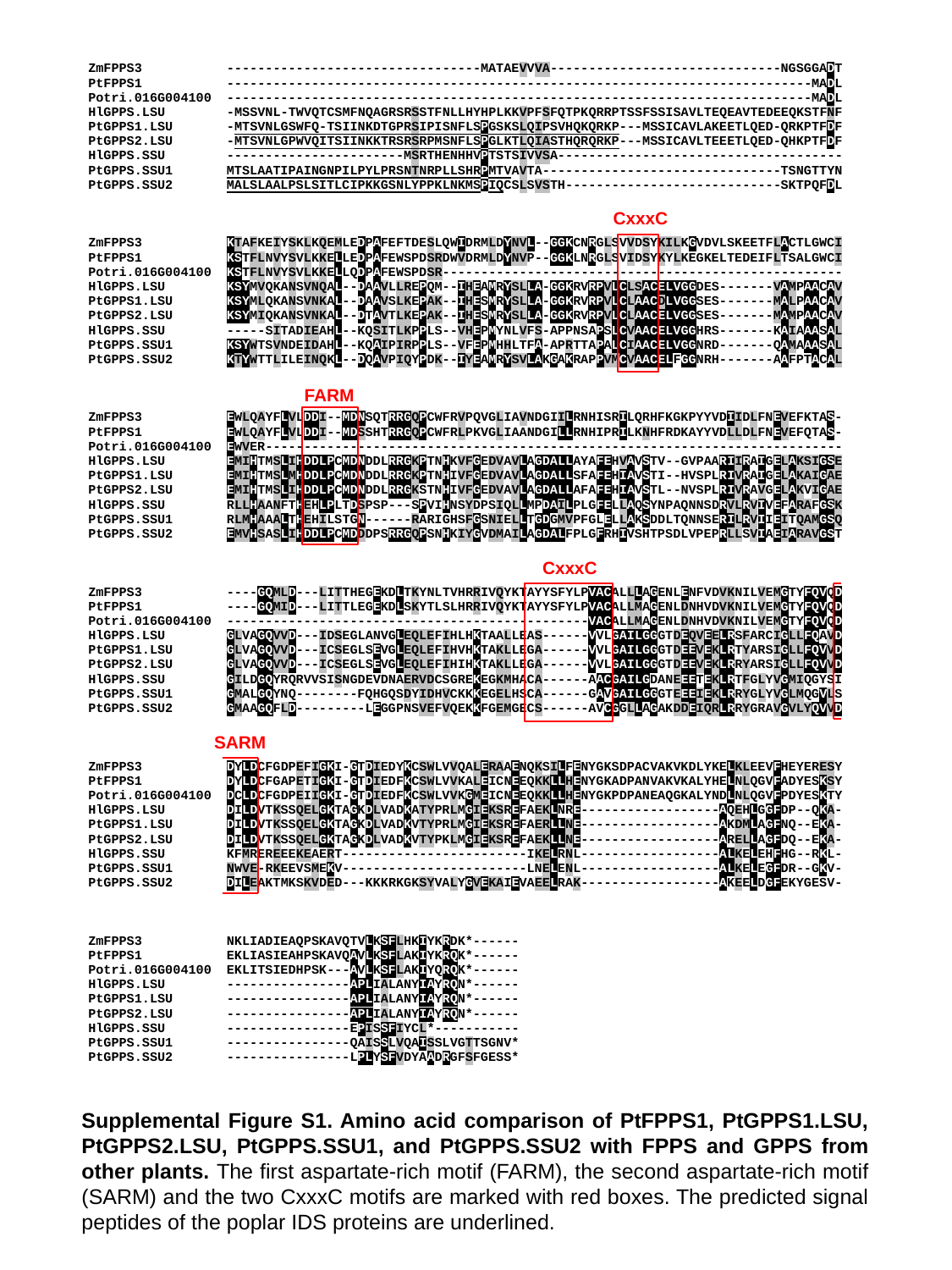

CxxxC
FARM
CxxxC
SARM
Supplemental Figure S1. Amino acid comparison of PtFPPS1, PtGPPS1.LSU, PtGPPS2.LSU, PtGPPS.SSU1, and PtGPPS.SSU2 with FPPS and GPPS from other plants. The first aspartate-rich motif (FARM), the second aspartate-rich motif (SARM) and the two CxxxC motifs are marked with red boxes. The predicted signal peptides of the poplar IDS proteins are underlined.

## Slide 2
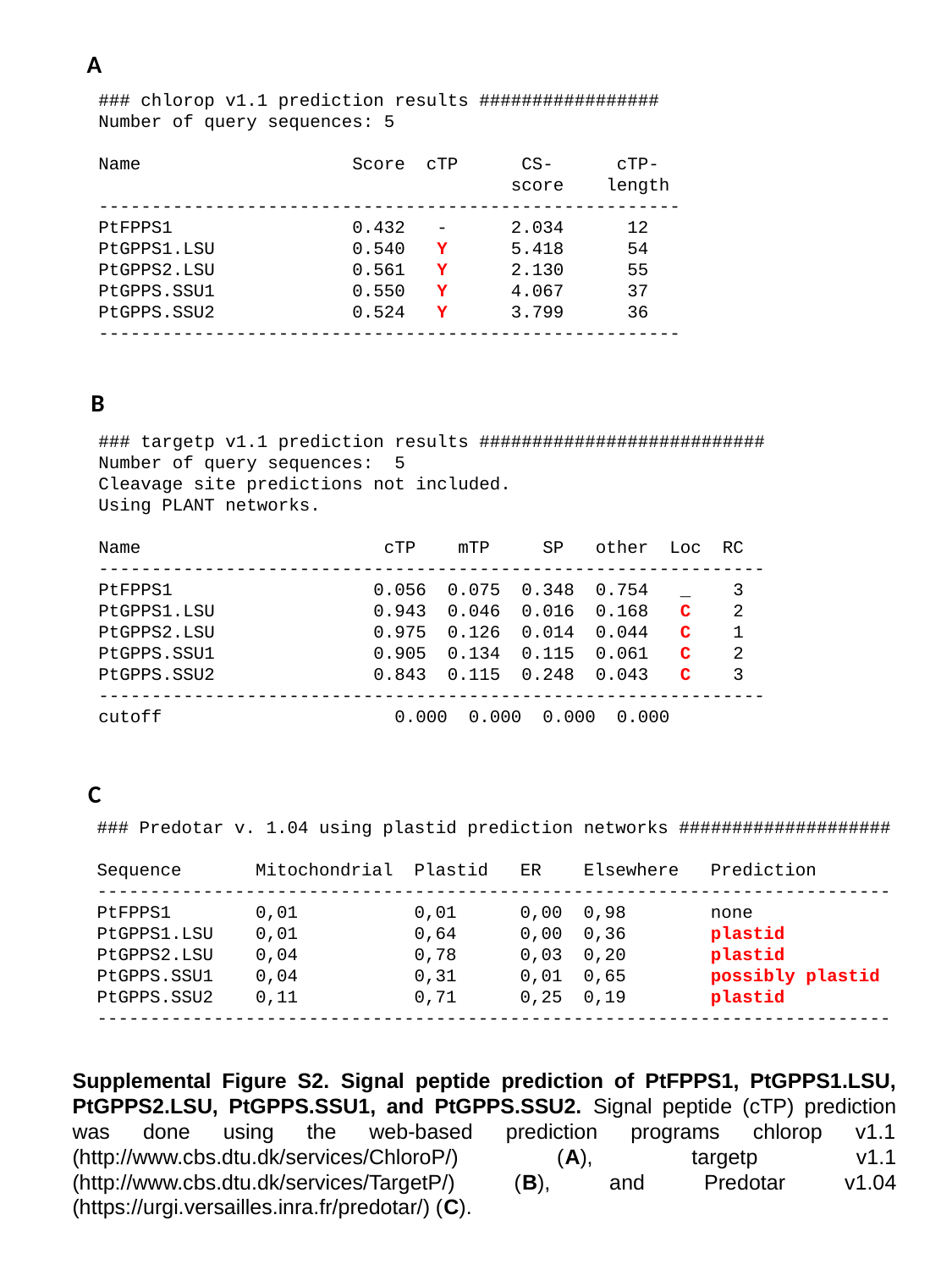

A
### chlorop v1.1 prediction results #################
Number of query sequences: 5
Name 		Score cTP CS- cTP-
 score length
-------------------------------------------------------
PtFPPS1 0.432 -	 2.034	 12
PtGPPS1.LSU 0.540 Y	 5.418	 54
PtGPPS2.LSU 0.561 Y	 2.130	 55
PtGPPS.SSU1 0.550 Y	 4.067	 37
PtGPPS.SSU2 0.524 Y	 3.799 36
-------------------------------------------------------
B
### targetp v1.1 prediction results ###########################
Number of query sequences: 5
Cleavage site predictions not included.
Using PLANT networks.
Name cTP mTP SP other Loc RC
---------------------------------------------------------------
PtFPPS1 0.056 0.075 0.348 0.754 _ 3
PtGPPS1.LSU 0.943 0.046 0.016 0.168 C 2
PtGPPS2.LSU 0.975 0.126 0.014 0.044 C 1
PtGPPS.SSU1 0.905 0.134 0.115 0.061 C 2
PtGPPS.SSU2 0.843 0.115 0.248 0.043 C 3
---------------------------------------------------------------
cutoff 0.000 0.000 0.000 0.000
C
### Predotar v. 1.04 using plastid prediction networks ####################
Sequence	 Mitochondrial Plastid ER Elsewhere Prediction
---------------------------------------------------------------------------
PtFPPS1 	 0,01	 0,01 0,00 0,98 none
PtGPPS1.LSU 0,01	 0,64 0,00 0,36 plastid
PtGPPS2.LSU 0,04	 0,78 0,03 0,20 plastid
PtGPPS.SSU1 0,04	 0,31 0,01 0,65 possibly plastid
PtGPPS.SSU2 0,11	 0,71 0,25 0,19 plastid
---------------------------------------------------------------------------
Supplemental Figure S2. Signal peptide prediction of PtFPPS1, PtGPPS1.LSU, PtGPPS2.LSU, PtGPPS.SSU1, and PtGPPS.SSU2. Signal peptide (cTP) prediction was done using the web-based prediction programs chlorop v1.1 (http://www.cbs.dtu.dk/services/ChloroP/) (A), targetp v1.1 (http://www.cbs.dtu.dk/services/TargetP/) (B), and Predotar v1.04 (https://urgi.versailles.inra.fr/predotar/) (C).

## Slide 3
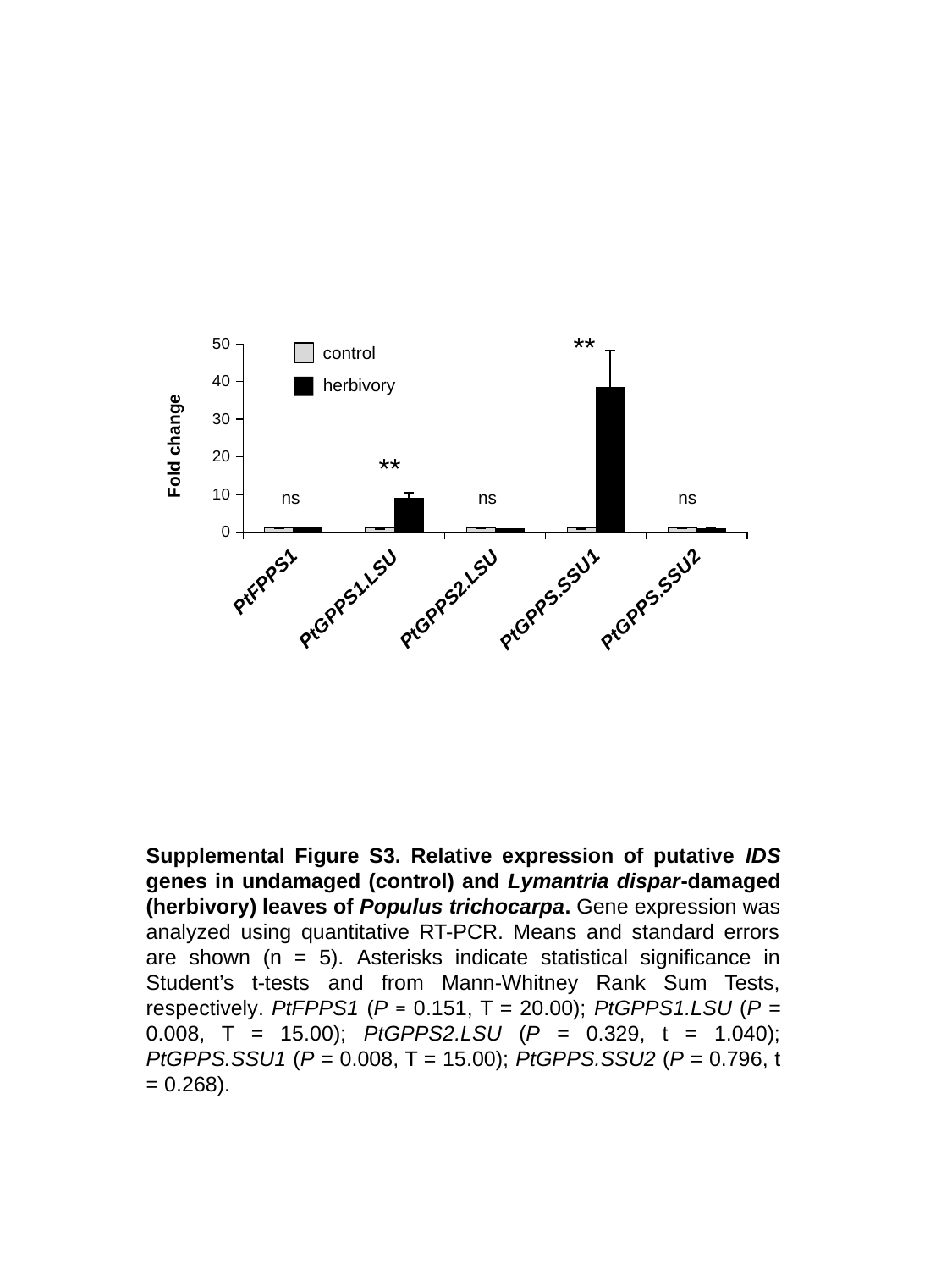

**
### Chart
| Category | | |
|---|---|---|
| PtFPPS1 | 1.0 | 1.0454545454545454 |
| PtGPPS1.LSU | 1.0 | 9.015481373971937 |
| PtGPPS2.LSU | 1.0 | 0.8245574725520949 |
| PtGPPS.SSU1 | 1.0 | 38.44574081665259 |
| PtGPPS.SSU2 | 1.0 | 0.9705279343926191 |control
herbivory
Fold change
**
ns
ns
ns
Supplemental Figure S3. Relative expression of putative IDS genes in undamaged (control) and Lymantria dispar-damaged (herbivory) leaves of Populus trichocarpa. Gene expression was analyzed using quantitative RT-PCR. Means and standard errors are shown (n = 5). Asterisks indicate statistical significance in Student’s t-tests and from Mann-Whitney Rank Sum Tests, respectively. PtFPPS1 (P = 0.151, T = 20.00); PtGPPS1.LSU (P = 0.008, T = 15.00); PtGPPS2.LSU (P = 0.329, t = 1.040); PtGPPS.SSU1 (P = 0.008, T = 15.00); PtGPPS.SSU2 (P = 0.796, t = 0.268).

## Slide 4
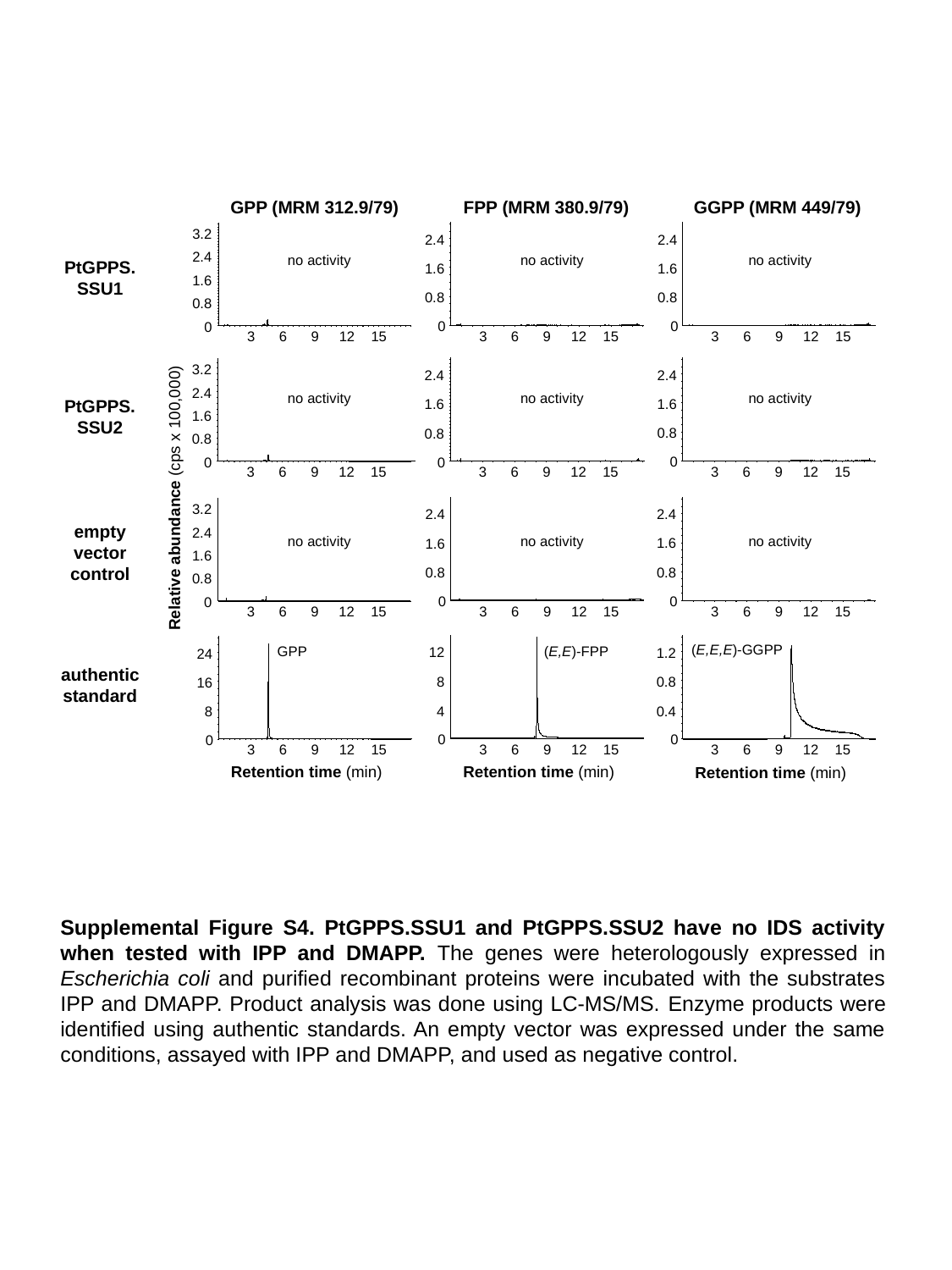

GPP (MRM 312.9/79)
FPP (MRM 380.9/79)
GGPP (MRM 449/79)
2.4
1.6
0.8
0
3
6
9
12
15
2.4
1.6
0.8
0
3
6
9
12
15
3.2
2.4
1.6
0.8
0
3
6
9
12
15
no activity
no activity
no activity
PtGPPS.
SSU1
2.4
1.6
0.8
0
3
6
9
12
15
2.4
1.6
0.8
0
3
6
9
12
15
3.2
2.4
1.6
0.8
0
3
6
9
12
15
no activity
no activity
no activity
PtGPPS.
SSU2
Relative abundance (cps x 100,000)
2.4
2.4
no activity
no activity
1.6
1.6
0.8
0.8
0
0
3
6
9
12
15
3
6
9
12
15
3.2
2.4
1.6
0.8
0
3
6
9
12
15
empty vector
control
no activity
(E,E,E)-GGPP
12
8
4
0
3
6
9
12
15
24
16
8
0
3
6
9
15
12
GPP
(E,E)-FPP
1.2
0.8
0.4
0
3
6
9
12
15
authentic
standard
Retention time (min)
Retention time (min)
Retention time (min)
Supplemental Figure S4. PtGPPS.SSU1 and PtGPPS.SSU2 have no IDS activity when tested with IPP and DMAPP. The genes were heterologously expressed in Escherichia coli and purified recombinant proteins were incubated with the substrates IPP and DMAPP. Product analysis was done using LC-MS/MS. Enzyme products were identified using authentic standards. An empty vector was expressed under the same conditions, assayed with IPP and DMAPP, and used as negative control.

## Slide 5
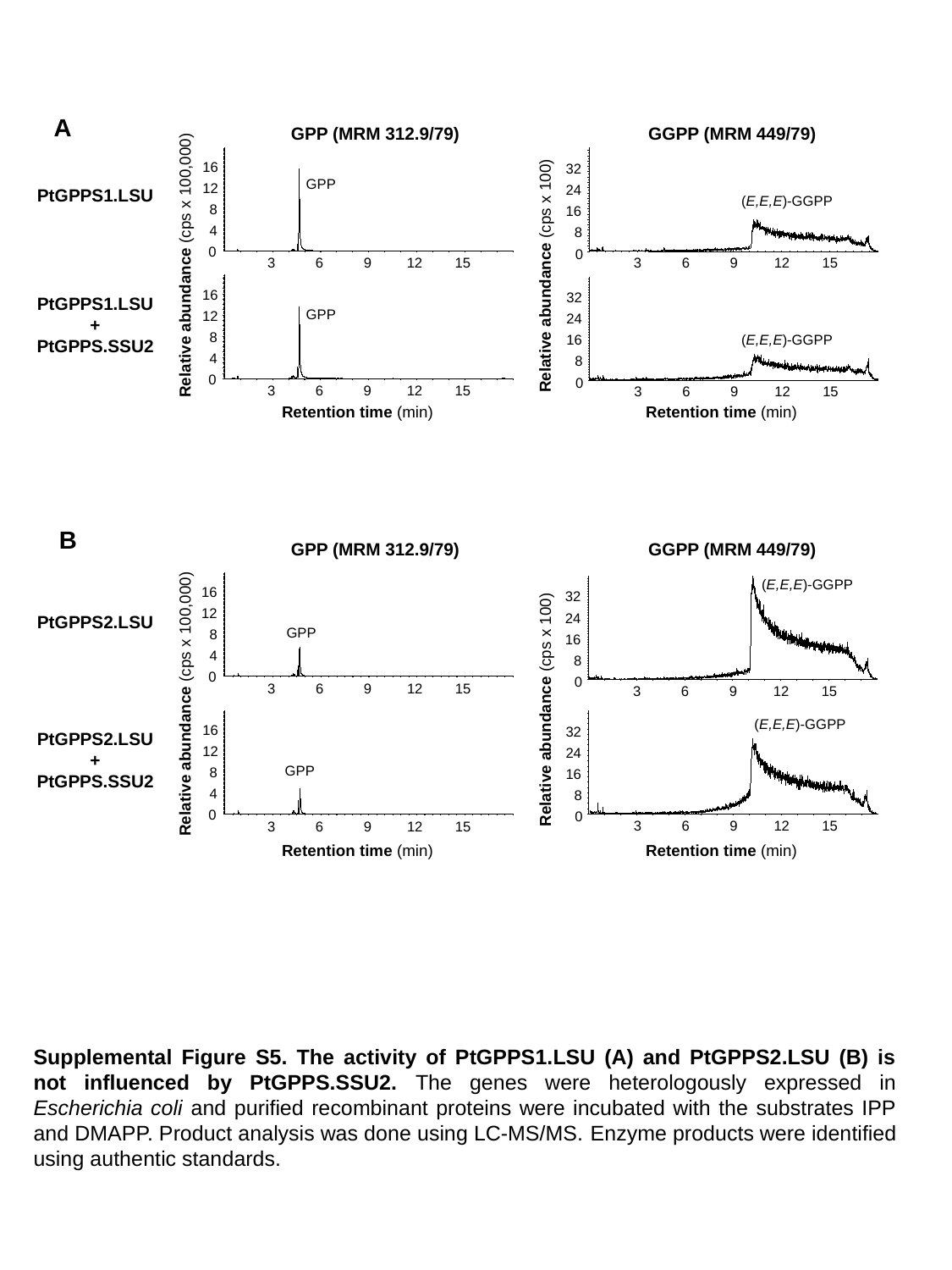

A
GPP (MRM 312.9/79)
GGPP (MRM 449/79)
16
12
8
4
0
3
6
9
12
15
32
24
16
8
0
3
6
9
12
15
GPP
PtGPPS1.LSU
(E,E,E)-GGPP
Relative abundance (cps x 100,000)
Relative abundance (cps x 100)
16
12
8
4
0
3
6
9
12
15
32
24
16
8
0
3
6
9
12
15
PtGPPS1.LSU
+
PtGPPS.SSU2
GPP
(E,E,E)-GGPP
Retention time (min)
Retention time (min)
B
GPP (MRM 312.9/79)
GGPP (MRM 449/79)
(E,E,E)-GGPP
16
12
8
4
0
3
6
9
12
15
32
24
16
8
0
3
6
9
12
15
PtGPPS2.LSU
GPP
Relative abundance (cps x 100,000)
Relative abundance (cps x 100)
(E,E,E)-GGPP
32
24
16
8
0
3
6
9
12
15
16
12
8
4
0
3
6
9
12
15
PtGPPS2.LSU
+
PtGPPS.SSU2
GPP
Retention time (min)
Retention time (min)
Supplemental Figure S5. The activity of PtGPPS1.LSU (A) and PtGPPS2.LSU (B) is not influenced by PtGPPS.SSU2. The genes were heterologously expressed in Escherichia coli and purified recombinant proteins were incubated with the substrates IPP and DMAPP. Product analysis was done using LC-MS/MS. Enzyme products were identified using authentic standards.
